# Pathogen Detection and Analysis of Non-Infectious Factors in Calf Diarrhea at a Large-Scale Dairy Farm in Wuwei City

**DOI:** 10.3390/vetsci12121150

**Published:** 2025-12-02

**Authors:** Yingjian Li, Lei Wang, Guowei Xu, Jingyan Zhang, Egide Hanyurwumutima, Yiduo Zhao, Jinhong Li, Xiaowei Feng, Yi Zhou, Ting Wang, Xiaoliang Chen, Lei Wei, Kang Zhang, Jianxi Li

**Affiliations:** Traditional Chinese Veterinary Technology Innovation Center of Gansu Province, Lanzhou Institute of Husbandry and Pharmaceutical Sciences, Chinese Academy of Agricultural Sciences, Lanzhou 730050, China; o264546465@163.com (Y.L.); wanglei03@caas.cn (L.W.); guoweixu1158@163.com (G.X.); zhangjingyan@caas.cn (J.Z.); wibekenda01@gmail.com (E.H.);

**Keywords:** calf diarrhea, pathogen detection, non-infectious factors

## Abstract

Given the high incidence of calf diarrhea and the limited data on pathogenic and non-infectious factors in a large-scale dairy farm in Wuwei City, this study aims to investigate the pathogens associated with summer calf diarrhea and the non-infectious risk factors in local large-scale dairy farm. It included 664 diarrheic calves (1–60 days of age, from June 2024 to May 2025), detected 5 types of enteric pathogens in 81 fecal samples collected in summer via RT-qPCR, collected data on non-infectious factors, and applied binary logistic regression and ROC analysis. The annual diarrhea incidence was 14.44%, with a peak in summer. *Clostridium perfringens* (67.90%) and Bovine Rotavirus (48.14%) were the dominant pathogens, and the rate of mixed infections was 48.14%. Risk factors for calf diarrhea included birth in spring or summer, female, Wagyu, younger age, birth weight < 38.5 kg, primiparous dams, serum immunoglobulin level < 8.1 g/L, and dam calving score > 1; longer duration of treatment was associated with a lower recovery rate. This study identifies key pathogens, risk factors, and their thresholds, thereby providing a scientific basis for the control of calf diarrhea in similar regions.

## Simple Summary

Calf diarrhea undermines calf health and consequently diminishes dairy farm profits on large dairy farm representing a key issue for the dairy industry. The purpose of this study was to identify the main pathogens associated with calf diarrhea during the peak season of diarrhea outbreaks and to analyze the association between diarrhea and various non-infectious factors, using a large dairy farm in Wuwei area as the research subject. Results showed annual diarrhea rate was 14.44% (summer highest); *Clostridium perfringens* was most common (67.90%), and nearly half the samples had multiple germs. Calves born in spring/summer, with low birth weight (under 38.5 kg), from first-time mothers, or with low immunoglobulin content (under 8.1 g/L) were more likely to get sick. Longer treatment meant lower recovery chances. These findings help farms here and similar areas prevent diarrhea, protecting calves and supporting dairy industry development.

## 1. Introduction

Calf diarrhea, or calf dysentery, is a common and serious condition, characterized by clinical signs such as dehydration, anorexia, and reduced mobility, all of which significantly compromise calf health and hinder growth [1]. Additional symptoms may include abnormal body temperature, tachycardia, and tachypnea [2]. Calf diarrhea increases the morbidity and mortality of neonatal calves, imposing a heavy economic burden on the livestock industry [3]. In China, the northwestern region serves as a core area for dairy cattle farming, where the combined prevalence rate of calf diarrhea reaches 25.2% [4]. Located in the “golden milk source belt”, Gansu Province has witnessed an increasingly prominent issue of calf diarrhea with the expansion of farming scale and the increase in breeding density, showing an incidence rate exceeding 20% [5]. This problem has become a critical bottleneck restricting Gansu’s transformation from a large cattle-farming province to a strong one.

The etiology of calf diarrhea can be divided into infectious and noninfectious factors. Infectious factors mainly include intestinal pathogens such as Bovine rotavirus (BRV), Enterotoxigenic *Escherichia coli* (ETEC), *Cryptosporidium* (COWP), and Bovine coronavirus (BCoV) [6]. Noninfectious factors include environmental influences, management practices, and physiological characteristics of the calves themselves. Among these, factors such as stocking density, hygiene of the calving area, feeding experience, and exposure to harmful environmental gases have been confirmed by multiple studies to contribute to the occurrence of diarrhea [7,8,9].

Wuwei City ranks as the largest city for dairy farming in Gansu Province. However, only one existing study has addressed single-pathogen infections associated with calf diarrhea in large-scale dairy farm in the Wuwei region, and the prevalence of mixed-pathogen infections during the peak incidence period remains poorly understood [10]. Meanwhile, the association between non-infectious factors and diarrhea has not been analyzed, making it difficult for local pastures to develop targeted prevention and control strategies. Therefore, this study focused on a large-scale dairy farm in the Wuwei region, aiming to identify the major pathogens associated with calf diarrhea during the high-incidence season and to analyze the relationships between diarrhea occurrence and various noninfectious factors, including season, sex, breed, week of age, birth weight, parity, calving conditions of the dam, and serum immunoglobulin concentrations. The findings of this study are expected to provide a scientific basis for establishing effective prevention and control strategies for calf diarrhea in this region and in other large-scale dairy operations under similar climatic and management conditions, thereby reducing the incidence of diarrhea and promoting the sustainable development of the local dairy industry.

## 2. Materials and Methods

### 2.1. Study Area and Management Practices

**Figure 1:**
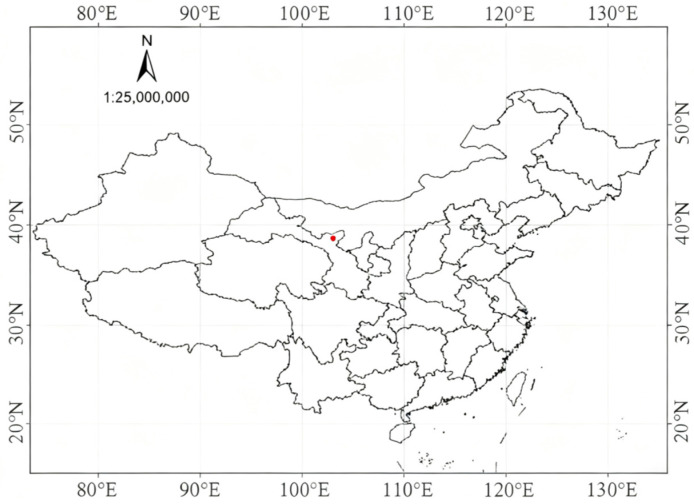
Geographical map of the dairy farm. The red dot represents the dairy farm surveyed.

This study was conducted on a large-scale dairy farm in the Wuwei region, with its geographical location shown in Figure 1. The farm employs a “4 + 2 + 0” colostrum feeding protocol for newborn calves: the first feeding of 4 L of colostrum occurs within 1 h after birth, followed by an additional 2 L feeding 2 h later, after which disbudding is performed. Calves are moved to a nursery pen within 20 min of birth and then transferred to individual calf hutches within 12–24 h. During the milk-feeding period, a staged whole milk feeding regimen is implemented: 2.5 kg/day from days 2–7, 3 kg/day from days 8–15, 6 kg/day from days 16–35, 5 kg/day from days 36–48, 4 kg/day from days 49–52, and 3 kg/day from days 53–60. From the second day after birth, calves have ad libitum access to starter feed and clean drinking water. The nutritional levels of the milk and starter feed are presented in Table 1.

### 2.2. Study Subjects and Sample Collection

The study subjects were calves aged 1–60 days, born on the farm between June 2024 and May 2025. The diagnostic criteria for calf diarrhea followed the method described by Riley et al. [11]. Basic data, including calf birth date, date of disease onset, sex, breed, dam parity, and dam calving ease score, were collected through the farm’s UniDairy management system. For 326 calves born in April 2025, 3 mL of venous blood was collected within 24 h after the first colostrum feeding. Serum was separated by centrifugation (3000 rmp, 10 min), and the Serum Total Protein (STP) level was measured using a DA7250 near-infrared analyzer. Serum STP level measured within 24–48 h after birth is reportedly highly correlated with colostral antibody absorption [12]. Therefore, STP concentration was used in this study to assess serum immunoglobulin levels. During the summer of 2025 (June–August), 77 rectal fecal samples (approximately 5 g each) were randomly collected from diarrheic calves within one month of age. The perianal area was cleaned and disinfected with alcohol swabs before sampling using sterile anal swabs. Samples were placed into sterile cryovials, stored in liquid nitrogen, and transported to the laboratory within 2 h for pathogen detection.

### 2.3. Principal Reagents and Instruments

Bacterial Genomic DNA Magnetic Beads Extraction Test Kit, Multi-5 Calf Diarrhea Pathogenic Microorganism Nucleic Acid Test Kit, and the Fluorescent Quantitative PCR Instrument were all purchased from Shenzhen EaseBio Technology Co., Ltd. (Shenzhen, China). The DA7250 Near-Infrared Analyzer was purchased from PentaBio Scientific Instruments (Beijing) Co., Ltd. (Beijing, China). The Centrifuge 5430 R, laminar flow cabinet, and metal bath heater were purchased from Eppendorf (Hamburg, Germany), Beijing Donglian Harl Instrument Manufacturing Co., Ltd. (Beijing, China), and Beijing Tongshihuagang Equipment Co., Ltd. (Beijing, China), respectively.

### 2.4. Pathogen Detection

From each fecal sample, 0.2 g was weighed and added to 1 mL of 0.9% sterile saline solution. The mixture was vortexed for 10 min to ensure thorough suspension, followed by centrifugation at 8000 rmp for 5 min. A 500 μL aliquot of the supernatant was collected for total nucleic acid extraction of pathogenic microorganisms using a commercial DNA extraction kit for animal pathogens, according to the manufacturer’s instructions. The extracted nucleic acids were stored at −20 °C until further analysis. Pathogens were detected using a multiplex calf diarrhea pathogen nucleic acid detection kit. The total reaction volume was 25 μL, consisting of 20 μL of multiplex reaction solution I or II and 5 μL of extracted nucleic acid template. Both negative and positive controls were included, with each control reaction containing 20 μL of the corresponding reaction solution and 5 μL of the negative or positive control template, respectively. Each control was tested in triplicate. The prepared reaction mixtures were subjected to quantitative real-time PCR using a fluorescence-based PCR instrument. The thermal cycling conditions were as follows: reverse transcription at 45 °C for 10 min (1 cycle), pre-denaturation at 95 °C for 3 min (1 cycle), followed by 40 cycles of denaturation at 95 °C for 10 s and annealing/extension at 60 °C for 30 s, during which fluorescence signals were collected. After amplification, the determination criteria refer to the instructions of the reagent kit.

### 2.5. Analysis of Non-Infectious Factors

The noninfectious factors included in this study were season (spring: March–May, summer: June–August, autumn: September–November, and winter: December–February), calf sex (male or female), breed (Holstein or Wagyu), week of age (1–9 wk), birth weight, dam parity, calving condition score, and serum immunoglobulin concentration of the calf. The calving condition score of the dam was evaluated according to the 5-point scale proposed by Hossein-Zadeh [13], as shown in Table 2. Calves that developed diarrhea between June 2024 and May 2025 were assigned to the diarrhea group. During the same period, all calves without diarrhea were identified from the UniDairy farm management system, and an equal number of calves were randomly selected using a random number table to serve as the control group. Differences in birth weight, dam parity, and calving condition score were compared between the two groups. Among the 326 calves born in April 2025, 56 calves with diarrhea were classified into the diarrhea group, while the remaining calves without diarrhea were used as the control group. The researchers compared the differences in serum immunoglobulin concentrations between the two groups. The week of diarrhea onset was recorded, and data on season, sex, and breed were collected for both diarrheic and healthy calves to analyze their associations with diarrhea occurrence. In addition, data on treatment duration and therapeutic outcomes in diarrheic calves were collected to evaluate the relationship between treatment duration and therapeutic outcome.

### 2.6. Data Analysis

All raw data were organized and screened for completeness and accuracy using Microsoft Excel 2016. Statistical analyses were conducted using IBM SPSS Statistics version 27 (IBM Corp., Armonk, NY, USA). The chi-square test was used to analyze the correlation between different seasons and diarrhea. A univariate binary logistic regression analysis was performed to investigate the potential association of breed and sex with calf diarrhea, with sex (female = 0, male = 1) and breed (Wagyu = 0, Holstein = 1) as the independent variables, and diarrhea status (absent = 0, present = 1) as the dependent variable. A univariate binary logistic regression analysis was employed to assess the correlation between treatment duration and therapeutic efficacy. Here, treatment duration served as the independent variable, while therapeutic outcome (coded as 0 = death, 1 = recovery) was defined as the dependent variable. The group coded as 0 was set as the reference group. Spearman’s rank correlation analysis was used to assess the association between week of age and the number of diarrhea cases, as well as the correlation between parity, maternal parturition status score, and immunoglobulin content (the correlation coefficient was denoted as r_s_), whereas Pearson’s correlation analysis was used to evaluate the correlation Analysis between birth weight and immunoglobulin content (the correlation coefficient is denoted by r). An independent sample *t*-test was used to compare the differences in birth weight, immunoglobulin content and other indicators between the diarrhea group and the control group. The results were expressed as “mean ± standard deviation”. The indicators with statistical significance were included in the multivariate binary logistic regression analysis to determine the independent influencing factors of calf diarrhea. The goodness-of-fit of the multivariate binary logistic regression model was verified by the Hosmer-Lemeshow test (*p* = 0.38 > 0.05), indicating no significant difference between observed and predicted values. Meanwhile, collinearity was tested using the variance inflation factor (VIF), and all variables had a VIF < 2, excluding the interference of multicollinearity. The risk effects of each factor were finally expressed as odds ratio (OR) and 95% confidence interval (95% CI). Receiver operating characteristic (ROC) curve analysis was used to determine the early warning thresholds of continuous factors such as birth weight and serum immunoglobulin concentration. The area under the curve (AUC) ranges from 0.5 to 1, and an AUC > 0.7 indicates that the factor has moderate or higher predictive value.

### 2.7. Ethical Approval

The animal handling protocol was approved by the Animal Ethics Committee, Lanzhou Institute of Animal Husbandry and Veterinary Pharmaceutics, Chinese Academy of Agricultural Sciences (Approval No: 2025-57) on 10 January 2025.

## 3. Results

### 3.1. Incidence of Calf Diarrhea

During the period from June 2024 to May 2025, a total of 664 calves developed diarrhea, corresponding to an average annual incidence rate of 14.44%. The number of cases recorded in spring, summer, autumn, and winter was 155, 208, 169, and 132, respectively (Table 3).

### 3.2. Pathogen Detection in Calves During Summer

**Figure 2:**
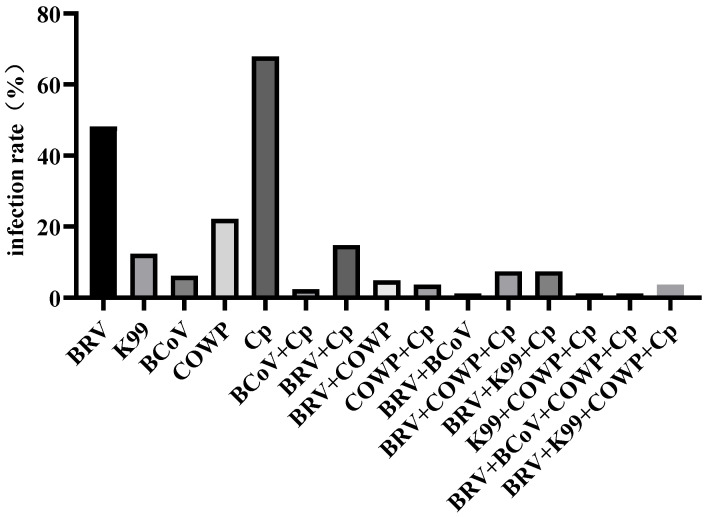
Bar chart of pathogen infection situation.

As diarrhea occurred most frequently during the summer, 81 diarrheic fecal samples collected in this season were tested for five major enteric pathogens: BRV, *Escherichia coli* K99 (K99), BCoV, COWP, and Cp. As shown in Table 4 and Figure 2, the positive detection rates were 48.14% (39/81) for BRV, 12.34% (10/81) for K99, 6.17% (5/81) for BCoV, 22.22% (18/81) for COWP, and 67.90% (55/81) for Cp. The overall mixed infection rate was 48.14% (39/81). Among the co-infections detected, the most common combinations were BRV + Cp (14.81%, 12/81), BRV + COWP + Cp, and BRV + K99 + Cp (both 7.40%, 6/81). Less frequent co-infections included BRV + COWP (4.93%, 4/81), COWP + Cp (3.70%, 3/81), BRV + K99 + COWP + Cp (3.70%, 3/81), BCoV + Cp (2.46%, 2/81), and BRV + BCoV, K99 + COWP + Cp, and BRV + BCoV + COWP + Cp (each 1.23%, 1/77). No pathogens were detected in 14 fecal samples. Overall, Cp and BRV were identified as the predominant pathogens during the summer season.

### 3.3. Correlation Between Season and Calf Diarrhea

The association between season and the risk of calf diarrhea was further analyzed. As shown in Table 5, no significant difference in diarrhea risk was observed between spring and summer (*χ*^2^ = 1.193, *p* = 0.275). The risk of diarrhea in spring was significantly higher than that in autumn (*χ*^2^ = 27.220, *p* < 0.01) and winter (*χ*^2^ = 5.798, *p* = 0.016). Similarly, the diarrhea risk in summer was significantly higher than in autumn (*χ*^2^ = 20.281, *p* < 0.01), but not significantly different from that in winter (χ^2^ = 2.310, *p* = 0.128). In addition, calves in autumn had a significantly higher risk of diarrhea than those in winter (*χ*^2^ = 6.523, *p* = 0.011). Overall, these results indicate that the incidence of calf diarrhea was markedly elevated during the spring and summer months compared with autumn and winter.

### 3.4. Association of Breed and Sex with Calf Diarrhea

**Figure 3:**
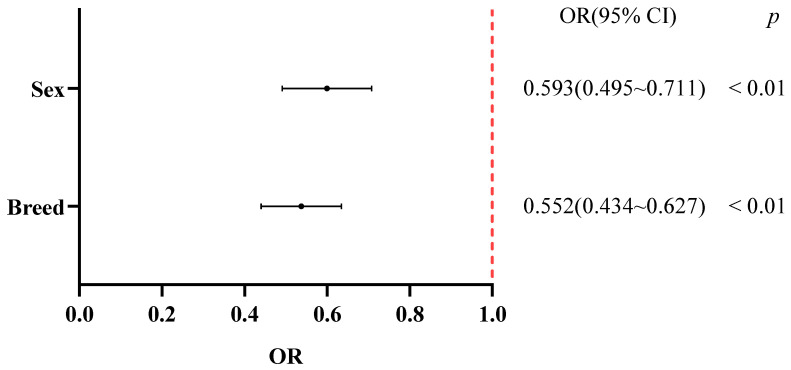
Forest plot of OR analysis by gender and breed.

As shown in Table 6 and Figure 3, both sex and breed were significantly associated with the incidence of calf diarrhea (*p* < 0.01). Male calves and Holstein calves were identified as independent protective factors against diarrhea.

### 3.5. Association Between Week of Age and Calf Diarrhea

**Figure 4:**
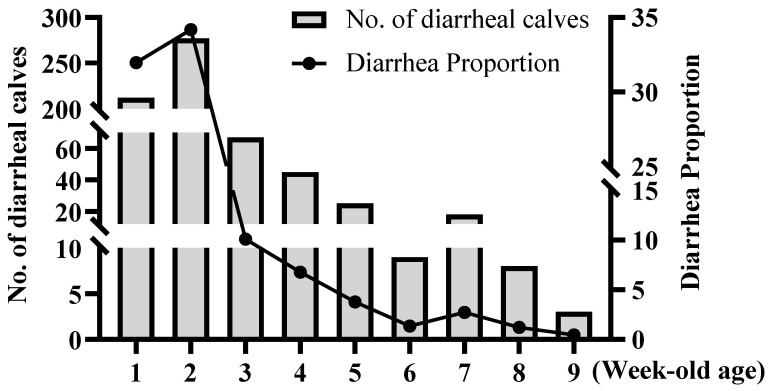
Data chart on diarrhea incidence among calves of different ages.

As shown in Table 7 and Figure 4, a highly significant negative correlation was observed between week of age and the number of diarrhea cases (*r_s_* = −0.967, *p* < 0.001). The highest number of diarrhea cases occurred at 2 weeks of age, with a total of 277 affected calves.

### 3.6. Association of Birth Weight, Parity, Calving Condition Score, and Serum Immunoglobulin Concentration with Calf Diarrhea

As shown in Table 8, extremely significant differences (*p* < 0.01) were observed between the Diarrhea and Control groups in birth weight, dam parity, serum immunoglobulin concentration, and calving condition score.

**Figure 5:**
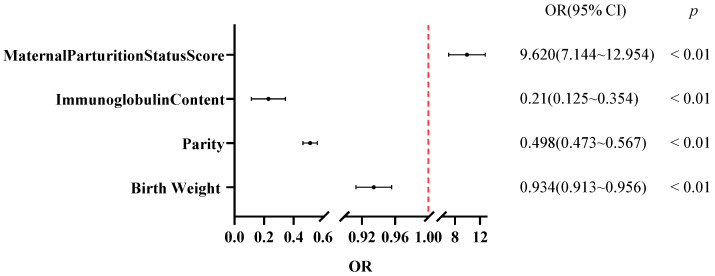
Forest plot of OR analysis for factors associated with calf diarrhea.

Using these variables as independent factors and diarrhea occurrence (no = 0, yes = 1) as the dependent variable, a multivariate binary logistic regression analysis was performed. The results (Table 9; Figure 5) showed that birth weight (*β* = −0.068, *p* < 0.01), parity (*β* = −0.698, *p* < 0.01), and serum immunoglobulin concentration (*β* = −1.559, *p* < 0.01) were highly significant negative predictors of diarrhea incidence, whereas calving condition score (*β* = 2.226, *p* < 0.01) was a highly significant positive predictor. These findings indicate that lower birth weight, lower parity, and lower immunoglobulin levels were independent protective factors against diarrhea, whereas a higher calving condition score represented an independent risk factor.

**Figure 6:**
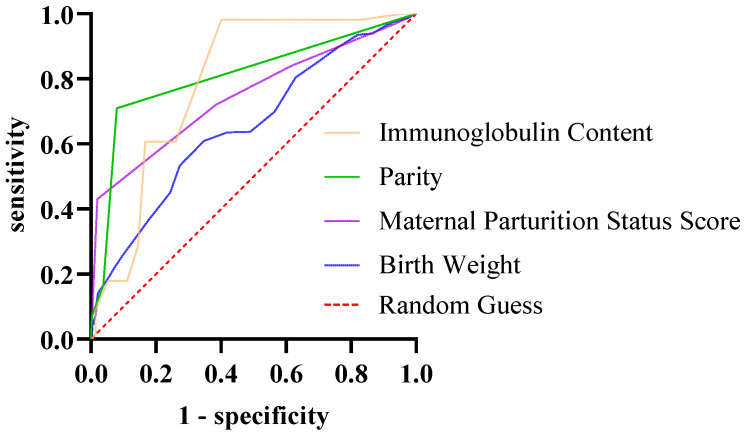
ROC curve for non-infectious factors.

ROC curve analysis was conducted to evaluate the diagnostic value of these factors (Table 10; Figure 6). The areas under the curve (AUC) for birth weight, parity, immunoglobulin concentration, and calving condition score were 0.6532, 0.7476, 0.7918, and 0.8091, respectively. Based on the Youden index, the optimal cutoff values for predicting diarrhea occurrence were 38.5 kg for birth weight, 1.5 for parity, 8.1 g/L for immunoglobulin concentration, and 1.5 for calving condition score. Consequently, calves with a birth weight < 38.5 kg, born to primiparous cows, with serum immunoglobulin concentrations < 8.1 g/L, or from dams with a calving condition score > 1 were more susceptible to diarrhea.

### 3.7. Correlation Among Birth Weight, Parity, Calving Condition Score, and Serum Immunoglobulin Concentration

As shown in Table 11, dam parity was highly and positively correlated with serum immunoglobulin concentration (*r* = 0.298, *p* < 0.01). No significant correlations were observed between serum immunoglobulin concentration and either birth weight or calving condition score (*p* > 0.05).

### 3.8. Association Between Treatment Duration and Therapeutic Outcome

A univariate binary logistic regression analysis was performed using treatment duration as the independent variable and therapeutic outcome (death = 0, recovery = 1) as the dependent variable. As shown in Table 12, treatment duration was highly and negatively associated with therapeutic outcome (*β* = −0.029, *p* < 0.01), indicating that calves requiring longer treatment periods had a significantly lower probability of recovery.

## 4. Discussion

Calf diarrhea is a multifactorial infectious syndrome affecting the gastrointestinal tract of calves and is a leading cause of high morbidity in dairy calves, significantly impacting their subsequent growth, development, and productive performance [14,15,16]. In this study, a survey conducted on a large-scale dairy farm in Wuwei, revealed an annual average incidence of calf diarrhea of 14.44%, indicating that this disease occurs frequently during farming and poses a substantial threat to calf health. Additionally, the results showed that summer is the peak season for diarrhea, likely associated with farm scale and management practices, which may further compromise calf immunity and increase susceptibility to secondary infections by pathogenic microorganisms, resulting in higher diarrhea incidence [7,8].

To investigate this phenomenon, 81 diarrheic fecal samples collected in summer were analyzed for five major pathogens using RT-qPCR. The results showed a high detection rate of Cp, 67.90%, while BRV, K99, COWP, and BCoV were detected at rates of 48.14%, 12.34%, 22.22%, and 6.17%, respectively, indicating that the outbreak was primarily caused by Cp infection, with frequent mixed infections. Previous studies have reported that Cp commonly spreads during cold seasons [17,18]; however, the high temperatures in Wuwei during summer may promote its proliferation [19]. Toxins produced by Cp, such as alpha-toxin and beta-toxin, can disrupt the intestinal mucosal barrier, resulting in intestinal wall edema, hemorrhage, and necrosis. This impairs the normal absorptive and secretory functions of the intestine, thereby causing diarrhea [6]. BRV also showed a relatively high detection rate. BRV infection can directly damage the epithelial cells of the intestinal villi, thereby reducing the absorptive surface area of the intestine. Unabsorbed substances such as glucose create a hyperosmotic environment that draws fluid into the intestinal lumen resulting in diarrhea. In addition, damage to the villous epithelial cells impairs local intestinal immune function, creating opportunities for secondary Cp infection [20]. A study has shown that diarrheic calves display intestinal dysbiosis characterized by a significant increase in the abundance of Enterobacteriaceae. When Cp and BRV co-infect the host, the α-toxin gene of C. perfringens and segment 6 dsRNA/VP7 gene of rotavirus may undergo synergistic amplification with *Enterobacteriaceae*, disrupting the intestinal barrier, and exacerbating the severity of diarrhea [21]. In contrast, BCoV had the lowest detection rate. As a conditional pathogen, BCoV rarely causes infection under good management conditions but can invade the host when environmental conditions deteriorate, with diarrhea usually occurring in calves aged 1–2 weeks [22,23]. The detection rate of COWP was relatively high. COWP is a major global cause of calf diarrhea [24], with multiple management factors influencing the risk of shedding [25,26], and its detection rate negatively correlates with age [27]. Notably, 48.14% of calves were co-infected with two or more pathogens. Mixed infections not only significantly increase diarrhea frequency but also exacerbate intestinal dysbiosis, mucosal inflammation, and electrolyte loss, thereby increasing mortality and complicating treatment [28].

Further multivariate statistical analysis identified several non-infectious risk factors contributing to diarrhea. Seasonal differences in diarrhea incidence were significant, with summer calves showing markedly higher risk than autumn calves, likely related to temperature and humidity, parasite lifecycles, and seasonal management practices. These findings are consistent with those reported by Svensson C and Park et al. [29]. Svensson C also noted that Swedish Red calves had significantly higher diarrhea risk than Swedish Holsteins. And this study also reached a similar conclusion regarding breed-related differences in diarrhea susceptibility—Wagyu calves were more susceptible than Holstein calves; however, the underlying causes of breed differences remain unclear. Studies suggest that breed-specific differences in infection tolerance are significant [30], leading us to speculate that Wagyu calves may have weaker immune responses and lower resistance to enteric pathogens compared with Swedish Holsteins.

In addition to season and breed, this study also identified sex as a significant factor influencing calf diarrhea. The risk of diarrhea was significantly lower in male calves than in females, a finding which contrasts with the results reported by Tikoo et al. [31]. One plausible explanation is that female calves are more susceptible due to differences in colostrum distribution and management [2].

Calf physiology also contributes to susceptibility. This study confirmed that the incidence of diarrhea peaks during the first and second weeks of life, with the highest risk occurring at two weeks of age consistent with the findings of Trotz-Williams LA and Park [29,32]. This period represents a critical “immunity gap” as calves transition from maternal antibody protection to active immunity. Insufficient intake of maternal antibodies, known as failure of passive immune transfer (FPT), leads to low immunoglobulin levels and significantly increases the risk [33]. Low immunoglobulin levels directly reflect passive immune transfer failure. Our study showed that immunoglobulin levels < 8.1 g/L were an extremely significant risk factor for diarrhea, consistent with findings by Bok M et al. [34]. Additionally, calves with birth weight < 38.5 kg were more prone to diarrhea, consistent with the range reported by Fagundes FT et al. [35]. Low birth weight may reflect weaker placental function and inadequate acquisition of maternal proteins and immunoglobulins, leading to insufficient passive immunity and poor nutrient reserves, thus increasing diarrhea susceptibility [36].

Maternal factors during gestation also influence calf diarrhea. This study systematically assessed the impact of maternal calving conditions and parity on offspring health for the first time in this region, revealing that dystocia (score > 1) significantly increased diarrhea risk. This aligns with the findings of Abebe R et al. [37]. Dystocia may also result in neonatal hypoxia, reduced vitality, and delayed colostrum intake, and adversely affect maternal production, reproduction, and health [38]. Parity was another significant factor, with first-parity calves being more prone to diarrhea, consistent with Silva-Del-Río N and Sutter F [39,40]. It is speculated that multiparous cows, having long-term antigen exposure, possess more developed immune systems and enhanced immunoglobulin G (IgG) synthesis in colostrum [41].

Analysis of treatment outcomes revealed a negative correlation between treatment duration and efficacy, suggesting that longer treatment was associated with poorer outcomes. On-site investigation identified suboptimal treatment and management practices: (1) prolonged antibiotic use, which is ineffective against viral infections like BRV and may induce resistance in bacteria such as Cp and K99; (2) limited use of oral electrolyte solutions, indicating insufficient supportive therapy; (3) reduced feeding of milk, milk replacer, or concentrates based on staff experience. Studies have shown that diarrheic calves retain sufficient digestive capacity to absorb milk [42].

To prevent Cp and BRV outbreaks during summer, vaccination of pregnant cows before the season is recommended to confer passive immunity to calves, reducing infection risk. During treatment, rapid pathogen detection using colloidal gold test strips on diarrheic fecal samples can guide therapy: viral infections should be managed primarily with oral electrolytes and traditional Chinese medicine [43], while bacterial infections can be treated with bacteriophage preparations [44,45,46]. The first 24 h after birth is a critical prevention window. For high-risk calves (birth weight < 38.5 kg, dystocia, first-parity, Wagyu breed, female), sufficient high-quality colostrum (IgG ≥ 20 g/L) should be fed within 4–6 h after birth, with a total intake of 4–5 L within 8 h. Colostrum can be pasteurized at 60 °C for 30–60 min to enhance IgG absorption and inactivate potential viruses. STP should be measured at 24–48 h; STP > 52 g/L indicates successful passive immunity transfer, while STP < 52 g/L requires supplemental high-quality colostrum or standardized colostrum replacers [12].

This study still has certain limitations: First, the research data were derived from a one-year observation in a single ranch. Although the sample size is sufficient, differences in climate and management models need to be considered when extrapolating the results, as these differences may restrict the scope of application of the risk thresholds. Second, other non-infectious risks were overlooked. The failure to incorporate quantitative analysis of environmental factors (such as fluctuations in temperature and humidity in calf barns, and bedding hygiene scores) may indirectly induce diarrhea by affecting intestinal barrier function. Therefore, future research can be advanced in two aspects: one is to conduct multi-center and cross-year studies covering ranches in different ecological regions such as Hexi Corridor and Longdong in Gansu Province, to verify the universality of risk thresholds and explore region-specific factors; the other is to integrate environmental monitoring and high-throughput sequencing technology, by recording microenvironmental parameters of calf barns and combining with 16S rRNA sequencing analysis of intestinal flora, to clarify how environmental factors indirectly induce diarrhea by affecting the function of the intestinal microbial barrier.

## 5. Conclusions

This study is the first to systematically characterize the pathogen profile and identify non-infectious risk factors for seasonal calf diarrhea on a large-scale dairy farm in the Wuwei region. It also establishes threshold values for these non-infectious factors locally. Based on these findings, a prevention and control strategy was developed, which can serve as a practical reference for scientifically managing calf diarrhea in this region and other farms under similar climatic and management conditions. This work has practical significance for reducing the incidence of calf diarrhea and promoting the sustainable development of the local dairy industry.

## Figures and Tables

**Table 1:** Nutrition levels of milk and starter feed.

Milk		Starter Feed	
Nutritional Level	Content (%)	Nutritional Level	Content (%)
Fat	4.55	CP	≥20.0
Milk protein	3.55	CF	≤13.0
Lactose	4.56	Ash	≤12.0
Non-fat milk solid	8.86	Ca	0.9–1.8
Whole milk solid	13.45	P	≥0.45
		NaCl	0.8–2.0
		Lys	≥0.65
		Moisture	≥14.0

**Table 2:** Scoring criteria for calving status of cows.

Score	Score Criteria
1	Normal, spontaneous delivery
2	Minor assistance, 1 person assisting traction
3	Moderate assistance, 2–3 persons assisting traction
4	Major assistance, use of calving forceps or internal delivery aids
5	Cesarean section or intrauterine calf extraction

**Table 3:** Incidence of calf diarrhea in dairy farm.

No. of Newborn Calves	No. of Diarrheal Calves	Diarrhea Rate	No. of Calves with Diarrhea by Season			
			Spring	Summer	Autumn	Winter
4608	664	14.44	155	208	169	132

**Table 4:** Pathogen infection status.

Agent	Positive Number	Sample Number	Infection Rate/(%)
BRV	39	81	48.14
K99	10	81	12.34
BCoV	5	81	6.17
COWP	18	81	22.22
Cp	55	81	67.90
BCoV + Cp	2	81	2.46
BRV + Cp	12	81	14.81
BRV + COWP	4	81	4.93
COWP + Cp	3	81	3.70
BRV + BCoV	1	81	1.23
BRV + COWP + Cp	6	81	7.40
BRV + K99 + Cp	6	81	7.40
K99 + COWP + Cp	1	81	1.23
BRV + BCoV + COWP + Cp	1	81	1.23
BRV + K99 + COWP + Cp	3	81	3.70
/	14	81	17.28

Note: “/” indicates “no pathogen detected”.

**Table 5:** Correlation analysis of calf diarrhea across different seasons.

Season	Spring		Summer		Autumn	
	*χ* ^2^	*p*	*χ* ^2^	*p*	*χ* ^2^	*p*
Spring						
Summer	1.193	0.275				
Autumn	27.220	<0.01	20.281	<0.01		
Winter	5.798	0.016	2.31	0.128	6.523	0.011

**Table 6:** Univariate binary logistic regression analysis table for variety and sex influencing factors.

Variable	*β*	SE	Wald *χ*^2^	OR	95% CI		*p*
					LL	UL	
Breed	−0.650	0.094	48.047	0.552	0.434	0.627	<0.01
Sex	−0.522	0.092	31.937	0.593	0.495	0.711	<0.01

**Table 7:** Statistical summary of diarrhea incidence among calves of different weeks of age.

Week of Age	No. of Diarrheal Calves	Diarrhea Proportion
1	212	31.96
2	277	34.18
3	67	10.09
4	45	6.77
5	25	3.76
6	9	1.35
7	18	2.71
8	8	1.20
9	3	0.45

**Table 8:** Univariate analysis of non-infectious factors associated with calf diarrhea.

Non-Infectious Factors	Diarrhea Group	Control Group	*t*	*p*
Birth Weight (kg)	36.84 ± 7.02	40.56 ± 5.76	10.56	<0.01
Parity	2.05 ± 1.18	3.06 ± 1.06	16.40	<0.01
Immunoglobulin Content (g/L)	7.61 ± 0.46	8.23 ± 0.63	20.46	<0.01
Maternal Parturition Status Score	1.95 ± 0.83	1.12 ± 0.44	22.74	<0.01

**Table 9:** Multivariate binary logistic regression analysis of non-infectious factors for calf diarrhea.

Non-Infectious Factors	*β*	SE	Wald *χ*^2^	OR	95% CI		*p*
					LL	UL	
Birth Weight (kg)	−0.068	0.012	34.067	0.934	0.913	0.956	<0.01
Parity	−0.698	0.066	110.334	0.498	0.473	0.567	<0.01
Immunoglobulin Content (g/L)	−1.559	0.266	34.393	0.21	0.125	0.354	<0.01
Maternal Parturition Status Score	2.226	0.152	222.331	9.620	7.144	12.954	<0.01

**Table 10:** ROC curve analysis table for non-infectious factors.

Non-Infectious Factors	AUC	Standard Error	Cutoff Point	95% CI		*p*
				LL	UL	
Birth Weight (kg)	0.6532	0.01490	38.5	0.6240	0.6824	<0.01
Parity	0.7476	0.01350	1.5	0.7211	0.7740	<0.01
Immunoglobulin Content (g/L)	0.7918	0.02707	8.1	0.7387	0.8448	<0.01
Maternal Parturition Status Score	0.8091	0.01254	1.5	0.7845	0.8337	<0.01

**Table 11:** Correlation analysis results between birth weight, parity, bitch birth condition score, and immunoglobulin content.

Item	Immunoglobulin Content (g/L)	
	*r*/*r_s_*	*p*
Birth Weight (kg)	0.04	0.467
Parity	0.298	<0.01
Maternal Parturition Status Score	0.052	0.346

**Table 12:** Results of univariate binary logistic regression analysis for treatment duration and treatment effectiveness.

Variable	*β*	SE	Wald *χ*^2^	OR	95% CI		*p*
					LL	UL	
Treatment Days	−0.029	0.011	7.182	0.972	0.952	0.992	<0.01

## Data Availability

The original contributions presented in this study are included in the article. Further inquiries can be directed to the corresponding author.

## Abbreviations

The following abbreviations are used in this manuscript:

BRV	Bovine rotavirus
BCoV	Bovine coronavirus
K99	*Escherichia coli* K99
COWP	*Cryptosporidium*
Cp	*Clostridium perfringens*
ETEC	Enterotoxigenic *Escherichia coli*
STP	Total Protein
ROC	Receiver operating characteristic
FPT	passive immune transfer
IgG	immunoglobulin G
CI	Confidence Interval
OR	Odds Ratio
AUC	area under the curve
VIF	Variance Inflation Factor
RT-qPCR	Reverse Transcription Quantitative Polymerase Chain Reaction
